# Association of relative deprivation with loneliness and its underlying mechanisms: Evidence from Chinese migrant children

**DOI:** 10.3389/fpsyg.2023.1048164

**Published:** 2023-03-09

**Authors:** Meng Xiong, Wenxi Xu

**Affiliations:** ^1^School of Education and Sports Sciences, Yangtze University, Jingzhou, China; ^2^Department of Psychology, University of Edinburgh, Edinburgh, United Kingdom

**Keywords:** belief in a just world, loneliness, migrant children, relative deprivation, self-esteem

## Abstract

Given the increase in the number of internal migrant children, the mental health problems (e.g., loneliness) of this population have received widespread attention. Relative deprivation is considered to be related to migrant children’s loneliness. However, the underlying mechanisms of this relationship remain unclear. Therefore, the present study tested the possible mediating role of self-esteem and the moderating role of belief in a just world in the association between relative deprivation and loneliness of migrant children. A total of 1,261 Chinese rural-to-urban migrant children (10–15 years old, *M*_age_ = 12.34 years, *SD* = 1.67; 52.0% males, 48.0% females; 23.55% fourth grade students, 16.49% fifth grade students, 19.59% sixth grade students, 15.54% seventh grade students, 13.80% eighth grade students, and 10.86% ninth grade students) were recruited to complete measures of relative deprivation, self-esteem, belief in a just world, loneliness, and demographic variables. Relative deprivation was significantly and positively correlated with migrant children’s loneliness, and this connection could be mediated by self-esteem. Moreover, the first part of the indirect effect of self-esteem on this link was moderated by belief in a just world. These effects were stronger for migrant children with higher levels of belief in a just world. This study reveals the potential mechanisms of relative deprivation affecting loneliness, while also providing insights into how to better help migrant children alleviate loneliness and improve their mental health.

## 1. Introduction

With economic, social, political, and technological development, the international migrant population has increased globally (International Organization for Migration, 2019). There are nearly 272 million international migrants worldwide, accounting for 3.5% of the world’s population (International Organization for Migration, 2019). Meanwhile, China’s migrant population has reached 376 million, accounting for 26% of the total population in 2021 (National Bureau of Statistics, PRC, 2021). Approximately 98.7 million children are migrants, accounting for 35.65% of the total number of children in China.

Based on China’s household registration (*hukou*) system, the children of migrant workers are considered temporary residents when they enter cities with their families. Therefore, they are not granted equal access to education and other social services in the cities where they live (Liu and Zhao, 2016). Such situations put migrant children at a disadvantage, and the social exclusion and unfair treatment they experience can significantly negatively impact their mental health (Fan et al., 2009, 2012; Euteneuer, 2014). In such an unjust and unfriendly social environment, migrant children are more likely to experience relative deprivation than urban children (Jin, 2016; Xiong et al., 2021a).

Relative deprivation is considered an important predictor of mental health problems (Demakakos et al., 2006; Kirkbride et al., 2014; Wickham et al., 2014). Previous studies have found that relative deprivation has a significant positive predictive effect on an individual’s mental health disorders, such as depression and anxiety (Eibner et al., 2004; Mishra and Carleton, 2015; Beshai et al., 2017). In the case of children, the influence of relative deprivation is more serious. A study of United States teens found that relative deprivation was significantly associated with loneliness; the higher the level of deprivation, the higher the risk of loneliness (Smith and Kessler, 2004). Loneliness is a common problem during teenage development (Alasmawi et al., 2020; Twenge et al., 2021); It comprises a painful experience where individuals’ social needs are not met (Wheeler et al., 1983; Cacioppo et al., 2006a). Loneliness not only affects physical health—e.g., increasing the incidence of cardiovascular disease (Caspi et al., 2006) and affecting individual immune function (Dixon et al., 2001)—but also has a serious impact on mental health—e.g., increasing depressive symptoms (Wei et al., 2005; Cacioppo et al., 2006b). In fact, loneliness is an indicator of mental illness (Hawkley and Cacioppo, 2010). Loneliness is even related to personality disorders and psychosis (Michalska da Rocha et al., 2018). Thus, although the association between relative deprivation and mental health issues (e.g., loneliness) has been confirmed, the mechanisms of this association need to be further explored (Creed and Reynolds, 2001).

Self-esteem is an important factor in the development of mental health in migrant children (Fan et al., 2012; Shoshani et al., 2014; Shen et al., 2015). Migrant children may experience a process of social re-adaptation when they move into a new city (Liu and Zhao, 2016), and self-esteem may affect their adaptation process (Portes and Zady, 2002; Berber Çelik and Odacı, 2020). On the one hand, some studies have focused on the negative correlation between relative deprivation and self-esteem (Lagacé, 2003; Tougas et al., 2004). On the other hand, existing research shows that self-esteem has a significant negative connection with loneliness in the new generation of migrant workers (Yang et al., 2011). Thus, self-esteem may mediate the relationship between relative deprivation and loneliness.

Moreover, previous studies have found that belief in a just world (BJW) helps maintain individuals’ mental health (Ritter et al., 1990; Dzuka and Dalbert, 2010; Jiang et al., 2016). Especially for disadvantaged or vulnerable groups, BJW is an important internal protective factor (Jiang et al., 2003; Zhang and Shen, 2011). Thus, it may also play a role in moderating the relationship between relative deprivation and self-esteem.

Therefore, we believe that it is necessary to clarify the relationship between relative deprivation and loneliness among Chinese migrant children and its potential mechanisms (e.g., the mediating role of self-esteem and the moderating role of BJW). We hope that this study can draw people’s attention to the relative poverty and loneliness of migrant children and promote people’s understanding of the effect of relative deprivation on loneliness and its underlying mechanisms. We hope that our findings can help migrant children and promote their mental health.

## 2. Theory and hypotheses

### 2.1. Relative deprivation and loneliness

As a powerful predictor of collective action, the sense of relative deprivation may influence collective behavior through perceptions of group weakness and injustice (Schulze and Krätschmer-Hahn, 2014). Relative deprivation refers to an individual or group’s perception of being disadvantaged, compared with a reference group; it does not originate from absolute disadvantage but from comparison with similar others (Runciman, 1966; Crosby, 1976; Walker and Smith, 2002).

According to social comparison theory, a person tends to evaluate their opinions, abilities, or behaviors by comparing with others like themselves (Festinger, 1954; Suls and Wheeler, 2000). Previous research has shown that through upward social comparison (i.e., when individuals compare themselves to those whom they believe to be better than them in some aspects), they may experience an increase in negative emotions (e.g., loneliness; Lim and Yang, 2019). Compared with urban children, migrant children are not granted equal access to education, medical care, and other social services in the cities where they live. Thus, many migrant children are excluded from public schools in cities. In the case of an uneven distribution of resources and opportunities, it is difficult to have a sense of belonging to the group and society (Chen and Feng, 2013). In such a negative social comparison environment, migrant children are more likely to develop a sense of relative deprivation, leading to mental health problems and loneliness (Fang et al., 2008). It is worth noting that migrant children’s loneliness is particularly serious (Kirova, 2001). Previous studies have shown that migrant children in China have a higher sense of loneliness than their non-migrant peers in the same city (Lu and Zhou, 2013; Liu et al., 2014).

Previous research has shown that relative deprivation is a significant predictor of poor physical and mental health (Gong et al., 2012; Elgar et al., 2013). Consistent with these theoretical notions, a few studies have found that relative deprivation can predict social loneliness (Silveira and Allebeck, 2001; Demakakos et al., 2006). Researchers have examined the link between relative deprivation and psychological well-being within migrant children and found that relative deprivation can make individuals feel isolated and hurt, especially in childhood (Valiente et al., 2010).

Based on the above, in this study, it is assumed that relative deprivation has a significant predictive effect on migrant children’s loneliness (H_1_).

### 2.2. The mediating role of self-esteem

The concept of self-esteem usually refers to a person’s evaluation of or attitude toward themselves. It also reflects an individual’s positive evaluation and experience of self-worth in the process of socialization (James, 1890; Rosenberg, 1965; Zhang and Li, 2009). Solomon et al. (2001) believe that self-esteem is the psychological mechanism individuals use to adapt to social and cultural environments, regulate their relationships with the environment, and affect the motivation and initiative of interpersonal communication.

Migrant children are discriminated against in the process of rural-to-urban migration and are considered temporary residents, making them susceptible to maladjustment (Fan et al., 2012; Hou et al., 2012; Shen et al., 2015). According to previous studies, self-esteem plays an important role in the social readjustment of migrant children (Gu et al., 2010; Thompson et al., 2016). It can effectively alleviate the psychological pressure, anxiety, and pain caused by discrimination, thereby maintaining the mental health of migrant children (Corning, 2002). Numerous studies suggest that migrant children with high self-esteem have fewer aggressive behaviors and negative emotions, which is more conducive to developing their mental health (Liang and Bian, 2011; Liu et al., 2020).

Given that self-esteem is a significant indicator of migrant children’s mental health (Mikkelsen et al., 2020; Caqueo-Urízar et al., 2021; Kuo et al., 2021), it may be related to their loneliness. According to the sociometer theory, self-esteem is the internal reaction of an individual’s interpersonal quality in a group; thus, when individuals are accepted, their self-esteem rises. In contrast, when an individual is rejected, the individual’s self-esteem declines. Therefore, poor self-esteem as a social meter sends out signals, causing negative emotions and increasing loneliness (Leary et al., 1995a; Leary, 2003; Leary, 2005; Zhang and Li, 2009). Studies on the direct link between self-esteem and loneliness have also verified that self-esteem is an important predictor of migrant children’s loneliness (Vanhalst et al., 2013; Kuo et al., 2021). For instance, numerous cross-sectional studies have documented a strong concurrent association between low self-esteem and loneliness (Mahon et al., 2006; Vanhalst et al., 2013; Zhao et al., 2013; Jiang and Shen, 2019). Especially in the case of teenagers, individuals with low levels of self-esteem may feel lonely (Dixon and Kurpius, 2008; Vanhalst et al., 2013). As a result, self-esteem has a significant predictive effect on the loneliness of migrant children.

Considering that self-esteem is one of the key factors influencing loneliness (Creemers et al., 2012; Kuo et al., 2021), many possible predictors of self-esteem have been considered. Numerous studies have revealed the predictive effects of perceived discrimination (Liu and Shen, 2010), peer victimization (Van Geel et al., 2018), parenting styles (Pinquart and Gerke, 2019), and social networking site use (Saiphoo et al., 2020) on self-esteem. However, for migrant children, the influence of relative deprivation on self-esteem needs to be further studied.

First, relative deprivation can significantly negatively impact the mental health, subjective well-being, and self-esteem of individuals, especially in the case of vulnerable groups (Xiong, 2015). For example, scholars have found that high relative deprivation may lead to psychosocial deficits, such as a lack of a sense of control, mastery over the environment, and self-esteem (Marmot et al., 1997; Eibner et al., 2004). These studies suggest that relative deprivation is negatively associated with self-esteem (Walker, 1999; Tougas et al., 2005). Second, the influence of relative deprivation on self-esteem also exists in this group (Petta and Walker, 1992; De La Sablonnière and Tougas, 2008). Some studies have shown a significant correlation between group relative deprivation and group self-esteem (Zagefka and Brown, 2005; De la Sablonniere et al., 2009). In addition, relative deprivation is an important factor affecting the mental health of migrant children (Cheung, 2014; Xiong, 2015). Migrant children are more likely to feel relative deprivation when they feel disadvantaged after social comparisons. This sense of relative deprivation may make it difficult for migrant children to develop a sense of belonging to urban children’s groups, which could negatively affect their self-esteem (Xiong, 2015; Xiong and Ye, 2016). Through unreasonable social comparison, unfair treatment has a major negative impact on the mental health, subjective well-being, and self-esteem of migrant children (Li, 2008; Kosaka et al., 2013).

Previous studies also found that self-esteem could mediate both the relationship between peer aggression and mental health problems (Ybrandt and Armelius, 2010), as well as the relationship between experiences of discrimination and loneliness (Świtaj et al., 2015). A prior study also demonstrated that self-esteem could mediate the link between relative deprivation and problematic mobile social media use (Yang and Jiang, 2020). Specifically, relative deprivation may lower the self-esteem of migrant children, thereby making them more inclined to view their relationship with the group negatively. Thus, failure to integrate into urban groups may be interpreted as rejection by migrant children with low self-esteem, and this rejection could contribute to increased feelings of loneliness.

Therefore, we assume that self-esteem mediates the association between relative deprivation and loneliness (H_2_).

### 2.3. The moderating role of belief in a just world

BJW refers to the idea that we live in a fair and orderly world where people get what they deserve (Lerner and Miller, 1978; Lerner, 1980). According to Dalbert’s (1999) viewpoint, BJW includes two dimensions: the general BJW and the individual BJW. The general BJW refers to a just environment where people get what they deserve, and the individual BJW refers to people convinced that the world is made just for them personally (Dalbert, 2001; Dalbert and Stoeber, 2005; Su et al., 2013). This belief that the environment is stable and orderly is conducive to an individual’s psychological and social adaptation. This allows individuals to believe that they can obtain the results they deserve by making efforts and following social norms (Hafer, 2000; Jiang et al., 2003; Laurin et al., 2011).

BJW serves as a guiding principle, suggesting that it helps migrant populations cope with social adaptation events by increasing feelings of confidence, control, and hope (Dzuka and Dalbert, 2010). Indeed, as a form of positive belief, BJW encourages people to see their social environment as more stable and controllable, which in turn may lower their perceptions of threat and increase their adaptability to urban groups and society (Dalbert, 1999; Furnham, 2003; Ramos et al., 2014). Studies have shown that people with high BJW are more capable of coping with negative events in their lives. Therefore, they report positive emotions and have high life satisfaction (Ritter et al., 1990; Dalbert, 2002; Zhang and Shen, 2011). Empirical research also supports that BJW is positively associated with individuals’ mental health (Ritter et al., 1990; Dalbert, 2002). For instance, a study conducted by Carifio and Nasser (2012) showed that individuals with higher BJW were better able to deal with stress stimuli and exhibit fewer symptoms of depression and anxiety than those with lower BJW. Thus, BJW helped people maintain their mental health and self-esteem. In conclusion, high BJW strengthens an individual’s emotional regulation ability and benefits their mental health.

Studies have also shown that BJW is more likely to facilitate well-being and self-esteem and enable people to better cope with hurts and injustice (Feather, 1991; Begue, 2005; Nie, 2015). People with high BJW tend to engage in cognitive reframing and suppress negative thoughts. In contrast, people with low BJW are vulnerable to feelings of injustice and have more negative emotions and poor self-esteem (Dalbert and Stoeber, 2006). Therefore, BJW can be seen as a personal resource that can protect one’s self-esteem (Jiang et al., 2017).

According to the justice motive theory, concern for justice is based on a “personal contract” between individuals and their social environment (Lerner, 1997; Dalbert, 2001). In developing their contract, BJW provides a psychological advantage by giving people the sense of a meaningful, predictable, and controllable life (Dalbert and Donat, 2015; Levesque, 2016). In particular, BJW alleviates the negative effects of adverse events on individuals’ physical and psychological development. It also protects people from the negative effects of worrying about the future and encourages individuals to pursue positive development (Hafer, 2000; Dalbert, 2001). Especially for vulnerable groups, BJW is an important internal protective factor (Zhang and Shen, 2011; Su et al., 2013). Therefore, empirical research has confirmed that high BJW contributes to the self-esteem of vulnerable groups and plays a role in moderating the relationship between relative deprivation and self-esteem (Jiang et al., 2017; Kim and Park, 2018; Lan et al., 2018).

Moreover, numerous studies indicate that BJW is an effective moderator (Osborne and Sibley, 2013; Xiong and Liu, 2020). For example, research has shown that BJW moderates the relationship between relative deprivation and depression in left-behind children (Xiong and Liu, 2020). Taken together, BJW may serve as a buffer in the relationship between relative deprivation and self-esteem.

Therefore, we hypothesize that BJW will moderate the relationship between relative deprivation and self-esteem in migrant children (H_3_).

### 2.4. The present study

Considering the impact of relative deprivation on the mental health of migrant children, especially when migrant children already experience severe loneliness (Lu and Zhou, 2013; Liu et al., 2014), it is vital to examine the mechanisms underlying the link between relative deprivation and loneliness in migrant children.

**Figure 1:**
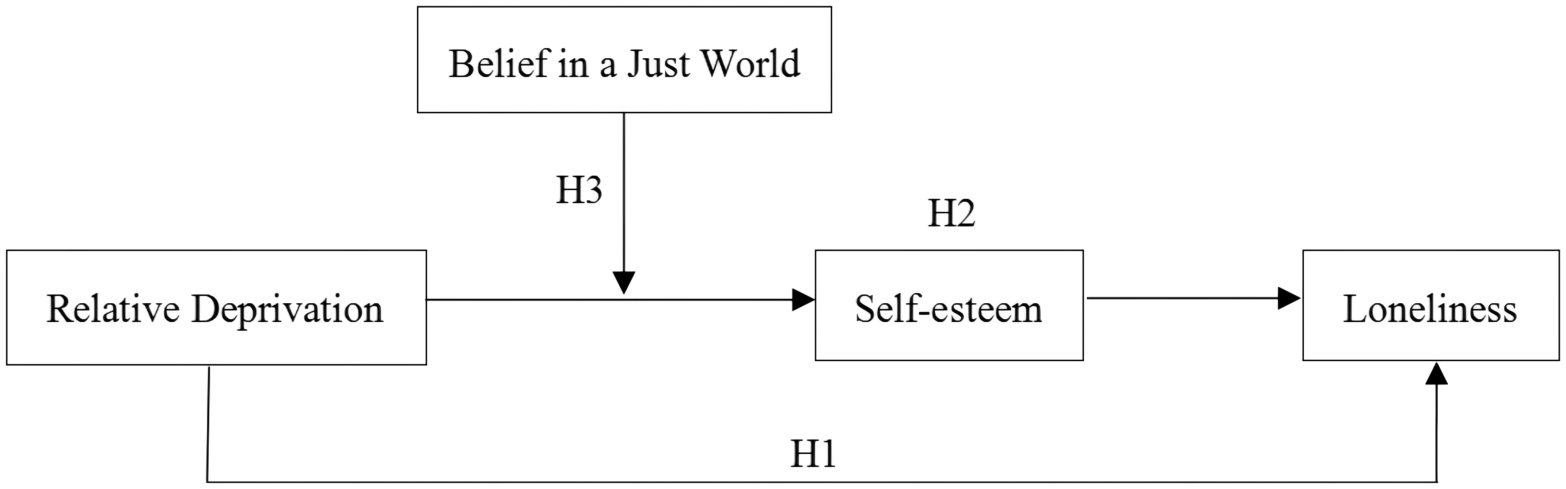
Moderated mediation model of the present study.

Given that self-esteem plays a bridging role in the relationship between relative deprivation and individuals’ mental health (Corning, 2002; Moradi and Subich, 2004; Seepersad, 2009), the current study attempts to examine the mediating effect of self-esteem on the association between relative deprivation and loneliness in migrant children. In addition, BJW has been considered an effective buffer to weaken relative deprivation and some risk factors for individuals’ mental health (Osborne and Sibley, 2013; Xiong and Liu, 2020). Therefore, BJW was tested as a moderator to reveal when the indirect relations between relative deprivation and loneliness are stronger or weaker. Figure 1 shows the proposed model.

## 3. Methods

### 3.1. Participants and procedures

Our sample consisted mainly of migrant children from three primary schools and three junior high schools in Fuzhou, Xiamen, Quanzhou, and other cities with large migrant populations (Xiong, 2015). It is worth noting that Fuzhou, Xiamen, and Quanzhou are the three most economically developed coastal cities in Fujian Province, and there are many migrant children in these areas. According to data from China’s Seventh Population Census, the migrant population in Fujian Province has reached almost 14 million, which accounts for 32.9% of the resident population. The eligibility criteria for migrant children were the following: (1) those born in rural regions, without urban *hukou* (permanent urban household registration) at birth; (2) those who accompanied their parents to the destination cities; and (3) those who had been living in their destination cities for more than 6 months (Chen et al., 2014).

The final sample included 1,261 migrant children. The mean age of the sample was 12.34 years (SD = 1.67); 52.0% (638 in total) were boys, and 48.0% (623 in total) were girls. Among them, 989 (78.43%) participants had siblings and 269 (21.33%) were only children (six participants did not report their only-child status). Additionally, 297 (23.55%) were in fourth grade, 208 (16.49%) in fifth grade, 247 (19.59%) in sixth grade, 196 (15.54%) in seventh grade, 174 (13.80%) in eighth grade, and 137 (10.86%) in ninth grade (two participants did not report their grade).

All questionnaires were pen and paper-based, and the method of cluster random sampling was adopted to recruit all participants. After obtaining informed consent from the teachers, participants, and the parents, we explained the instructions to the participants. Those in the target groups were invited to participate anonymously in the classroom. The authenticity, independence, and integral nature of all answers and the confidentiality of the information collected were emphasized to all participants by well-trained psychology graduate students. Data collection took ~30 min. All students received a ballpoint pen as a reward after completing the survey. To ensure the quality of the questionnaire data, the following procedures were instituted. First, if more than three items were left unanswered or answered in the same way, the responses were deleted. Second, for the remaining questionnaires, the mean method was adopted to handle missing values. Finally, 1,261 questionnaires were validated, which accounted for 97.75% of the total administered.

### 3.2. Measures

#### 3.2.1. Relative deprivation

Relative deprivation was measured using the Relative Deprivation Scale for Migrant Children (Ye and Xiong, 2017). The scale includes four dimensions: cognition of individual relative deprivation [composite reliability (CR) = 0.71], emotion of individual relative deprivation (CR = 0.81), cognition of group relative deprivation (CR = 0.87), and emotion of group relative deprivation (CR = 0.89). The scale consists of 20 items (e.g., “*What do you think of your family’s economic status, compared with that of your urban counterparts*?”) measuring five aspects of migrant children’s current situation (e.g., family economic status, housing conditions, residential stability, development of strong points, and parental involvement in education). The items range from 1 (“very good”) to 7 (“very bad”) for the cognitive dimension, and from 1 (“very satisfied”) to 7 (“extremely unsatisfied”) for the emotional dimension. Higher scores indicate higher levels of relative deprivation. The items demonstrated high reliability and validity in the present study (Cronbach’s *α* = 0.92, *χ*^2^/*df* = 4.85, CFI = 0.96, TLI = 0.95, RMSEA = 0.06).

#### 3.2.2. Belief In a just world

BJW was measured using the BJW Scale (Dalbert, 1999; Su et al., 2012). The scale consists of two subscales with 13 items, named as personal BJW (CR = 0.85, e.g., “*I believe that most things that happen in my life are just*”) and general BJW (CR = 0.78, e.g., “*In the long run, I believe that those who suffer injustice will be compensated*”). Participants responded on a five-point Likert-type scale ranging from 1 (“strongly disagree”) to 5 (“strongly agree”). Cronbach’s α for the scale was 0.88. The reliability coefficients were 0.85 for personal BJW and 0.79 for a general BJW. We averaged the scores of all items from these two subscales to measure BJW, with higher scores representing higher levels of BJW. Tests indicated that the items have good validity (*χ*^2^/*df* = 4.73, CFI = 0.96, TLI = 0.95, RMSEA = 0.05).

#### 3.2.3. Self-esteem

The Chinese version of the 10-item Rosenberg Self-Esteem Scale (Rosenberg, 1965) was adopted to measure self-esteem. For each item (e.g., “*I feel that I have many good qualities*”), participants responded on a four-point Likert-type scale ranging from 1 (“strongly disagree”) to 4 (“strongly agree”). Higher scores indicated higher self-esteem. This scale has been used with good reliability and validity in a sample of Chinese migrant children (Xiong and Liu, 2020). In the current study, the items demonstrated good internal consistency and construct validity (Cronbach’s α = 0.80, *χ*^2^/*df* = 4.42, CFI = 0.97, TLI = 0.95, RMSEA = 0.05; CR = 0.77).

#### 3.2.4. Loneliness

The loneliness of migrant children was assessed using the Adolescence Loneliness Scale (Zou, 2003). Twenty-one items were used to measure pure loneliness (CR = 0.85, e.g., “*I often feel rejected by my classmates*”), perception of one’s social skills (CR = 0.82, e.g., “*I make friends easily at school*”), evaluation of current relationships (CR = 0.76, e.g., “*I am always a loner in class*”), and perception of unmet needs in important relationships (CR = 0.73, e.g., “*I have no one to talk to in class*”) on a scale from 1 (“not at all”) to 4 (“fully complete”). Higher scores represented more serious psychological distress. Originally, high scores of social ability perception represented a positive evaluation, and high scores of the other three dimensions represented negative evaluations. The dimension of social ability perception was thus reversed and added to the other three-dimension scores, while its average score was taken as the total loneliness score; the higher the score, the stronger the loneliness. In this study, the internal consistency coefficient of the questionnaire was 0.92. The scale showed good validity (*χ*^2^/*df* = 4.89, CFI = 0.94, TLI = 0.93, RMSEA = 0.06).

### 3.3. Data analysis

Previous studies have shown that age, gender, one-child status, and grade affect individual loneliness (Hackett et al., 2012; Cole et al., 2021; Lin et al., 2021). Therefore, these demographic variables were included as covariates in all models in the present study.

First, IBM SPSS 26 and AMOS 24 software were used to test the reliability and validity of the scales used (see Table 1). Second, IBM SPSS 26 was used for descriptive statistics and correlation analysis. Third, we used analyses of variance and post-test (least significant difference [LSD]) to explore differences in loneliness among demographic variables. Fourth, according to the moderated mediation effect analysis procedure recommended by Wen and Ye (2014), using Mplus software (which can obtain all the required results at one time, including the usual sequential test results and the bootstrap confidence interval), under the condition of controlling core demographic variables, the moderated mediation model was tested by adopting the nonparametric percentile bootstrap method with deviation correction. In order to further confirm the accuracy of the research results, the method of replacing the econometric model and reducing the sample size was used to test the robustness of the relationship between relative deprivation and loneliness. Moreover, in order to confirm whether the result of using the imputed sample (*N* = 1,261) is consistent with the result of using the unimputed sample, a robustness test was also conducted based on the unimputed sample (*N* = 773). Fifth, Harman’s one factor test, consisting of a preliminary factor analysis comprising all the items included in the questionnaire, was performed (Zhou and Long, 2004). In this study, 12 factors emerged from the factor analysis, while the first factor explained only 20% of the variance. This shows that the common method variance was restricted in our data. Finally, confirmatory factor analysis showed that the four-factor model in this study showed a very good fit (*χ*^2^/*df* = 3.18; CFI = 0.99; TLI = 0.99; RMSEA = 0.04; SRMR = 0.01), confirming the structural validity of the measurement model.

**Table 1:** Scale dimensions reliability and validity.

Scale	Dimension	Cronbach’s *α*	CR	*χ*^2^/*df*	CFI	TLI	RMSEA
Relative deprivation		0.92		4.85	0.96	0.95	0.06
	Cognition of individual relative deprivation	0.70	0.71				
	The emotion of individual relative deprivation	0.80	0.81				
	Cognition of group relative deprivation	0.88	0.87				
	The emotion of group relative deprivation	0.83	0.89				
Belief in a just world		0.88		4.73	0.96	0.95	0.05
	Personal belief in a just world	0.85	0.85				
	General belief in a just world	0.79	0.78				
Self-esteem		0.80	0.77	4.42	0.97	0.95	0.05
Loneliness		0.92		4.89	0.94	0.93	0.06
	Pure loneliness	0.85	0.85				
	Perception of one’s social skills	0.81	0.82				
	Evaluation of current relationships	0.77	0.76				
	Perception of unmet needs in important relationships	0.75	0.73				

*N* = 1,261. CR = composite reliability.

## 4. Results

### 4.1. Preliminary analysis

**Figure 2:**
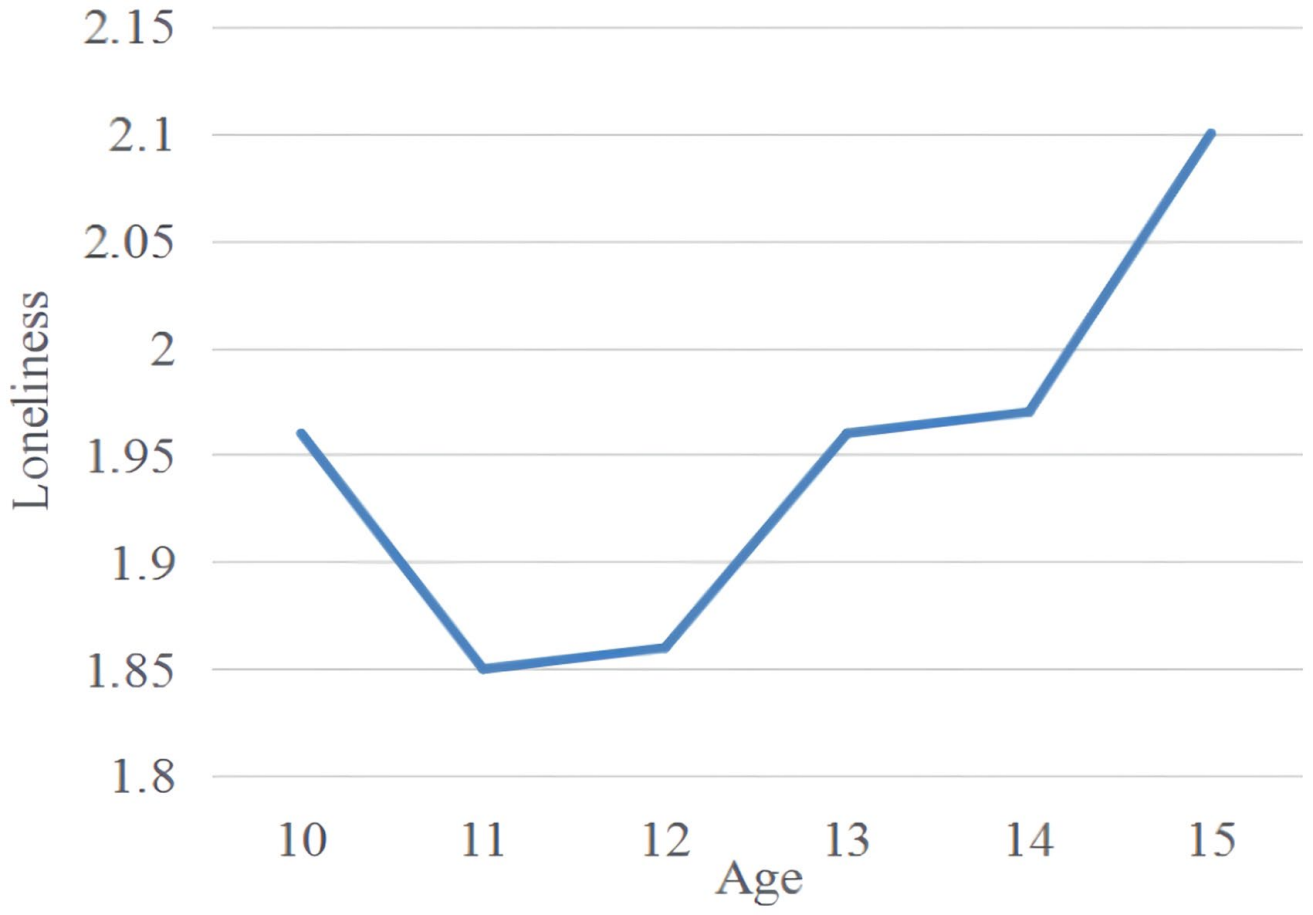
A post-test (LSD) of age differences in loneliness.

Table 2 presents the means, standard deviations, and correlations among relative deprivation, loneliness, self-esteem, and BJW. Among them, the means of relative deprivation, BJW, loneliness, and self-esteem were 3.24 (SD = 0.95), 4.17 (SD = 0.94), 1.94 (SD = 0.60), and 2.88 (SD = 0.50), respectively. As hypothesized, relative deprivation was positively correlated with loneliness and negatively correlated with self-esteem and BJW. Self-esteem was negatively correlated with loneliness and positively correlated with BJW. BJW was negatively correlated with relative deprivation. Additionally, in a one-way analysis of variance, there were significant differences in loneliness according to subjects’ age (*F* [5,1255] = 4.00, *p* < 0.05), and there were no significant differences in loneliness according to subjects’ gender (*F* [1,1259] = 1.57, *p* > 0.05), only-child status (*F* [2,1258] = 1.65, *p* > 0.05), and grade (*F* [6,1254] = 1.73, *p* > 0.05). Meanwhile, according to post-test (LSD), the loneliness of 15-year-old migrant children was significantly higher than that of other ages. Overall, there was a general upward trend in loneliness with age (see Figure 2).

**Table 2:** Correlation statistical analyses.

Variable	1	2	3	4	5	6	7	8
1. Age	–							
2. Gender	−0.01	–						
3. Only-child status	0.02	−0.05	–					
4. Grade	0.16^**^	−0.02	−0.03	–				
5. Relative deprivation	0.22^**^	−0.03	0.00	0.25^**^	–			
6. Belief in a just world	−0.19^**^	0.03	0.02	−0.71^**^	−0.39^**^	–		
7. Loneliness	0.08^**^	−0.04	0.01	0.24^**^	0.24^**^	−0.35^**^	–	
8. Self-esteem	−0.07^**^	0.05	0.02	−0.28^**^	−0.27^**^	0.37^**^	−0.47^**^	–

*N* = 1,261. ^*^p < 0.05, ^**^p < 0.01, ^***^p < 0.001.

### 4.2. Mediating effect of self-esteem

We conducted a mediation analysis to test whether self-esteem mediated the effect of relative deprivation on loneliness in migrant children (see the model in Figure 1). All variables were standardized. Bootstrap estimates were based on 5,000 bootstrap samples. Table 3 presents the results of the statistical mediation. When controlling for age, gender, only-child status, and grade, relative deprivation negatively predicted self-esteem (*B* = −0.22, *t* = −7.73, *p* < 0.001), and self-esteem negatively predicted loneliness (*B* = −0.42, *t* = −15.97, *p* < 0.001). Meanwhile, the direct relationship between relative deprivation and loneliness was also significant (*B* = 0.11, *t* = 3.97, *p* < 0.001). The bias-corrected percentile bootstrap method indicated that the mediating effect of self-esteem was 0.09, and its 95% confidence interval did not contain 0 ([0.06, 0.12]). This indirect effect accounted for 46.06% of the total effect. Thus, Hypothesis 2 was supported.

**Table 3:** Testing the mediation effect of self-esteem.

Outcome (*Y*)	Predictors (*X*)	Model summary			*B*	SE	95% CI
		*R*	*R* ^2^	*F*			
Loneliness		0.30	0.09	25.08^***^			
	Age				0.00	0.02	[−0.03, 0.03]
	Gender				−0.05	0.05	[−0.16, 0.05]
	Only-child status				0.03	0.07	[−0.10, 0.16]
	Grade				0.11^***^	0.02	[0.08, 0.14]
	RD				0.20^***^	0.03	[0.14, 0.25]
Self-esteem		0.35	0.12	35.44^***^			
	Age				−0.01	0.02	[−0.03, 0.04]
	Gender				0.09	0.05	[−0.02, 0.19]
	Only-child status				0.02	0.07	[−0.11, 0.15]
	Grade				−0.14^***^	0.02	[−0.17, −0.11]
	RD				−0.22^***^	0.03	[−0.27, −0.16]
Loneliness		0.49	0.24	67.63^***^			
	Age				0.00	0.02	[−0.03, 0.03]
	Gender				−0.02	0.05	[−0.11, 0.08]
	Only-child status				0.04	0.06	[−0.08, 0.16]
	Grade				0.05^***^	0.02	[0.02, 0.08]
	RD				0.11^***^	0.03	[0.05, 0.16]
	Self-esteem				−0.42^***^	0.03	[−0.47, −0.37]
**Effect**	***B***	**Boot SE**		**Boot LLCI**		**Boot ULCI**	
Direct	0.11	0.03		0.00		0.05	
Indirect	0.09	0.01		0.06		0.12	

*N* = 1,261. RD, relative deprivation. Bootstrap sample size = 5,000. LL, low limit; CI, confidence interval; UL, upper limit. ^*^p < 0.05, ^**^p < 0.01, ^***^p < 0.001.

### 4.3. Moderating effect of BJW

We examined whether the strength of the mediated relationships was contingent on BJW. Specifically, we tested whether BJW moderated the effect of relative deprivation on self-esteem in the mediation models (see the model in Figure 1). The results of the analysis are presented in Table 4. After controlling for age, gender, only-child status, and grade, a significant effect of relative deprivation on self-esteem was observed (*B* = −0.59, *t* = −28.28, *p* < 0.001), and this effect was moderated by BJW (*B* = 0.23, *t* = 24.52, *p* < 0.001).

**Table 4:** Moderated mediation analysis results with belief in a just world as a moderator.

Outcome (*Y*)	Predictors (*X*)	Model summary			*B*	SE	95% CI
		*R*	*R* ^2^	*F*			
Self-esteem		0.40	0.16	34.91^***^			
	Age				0.06^***^	0.02	[0.02, 0.09]
	Gender				0.01	0.01	[−0.01, 0.03]
	Only-child status				−0.10^***^	0.03	[−0.17, −0.05]
	Grade				−0.18^***^	0.02	[−0.22, −0.15]
	RD				−0.59^***^	0.04	[−0.66, −0.52]
	BJW				−0.04	0.03	[−0.09, 0.01]
	RD × BJW				0.23^***^	0.04	[0.19, 0.28]
Loneliness		0.49	0.24	67.63^***^			
	Age				−0.04	0.03	[−0.10, 0.02]
	Gender				−0.03	0.03	[−0.09, 0.02]
	Only-child status				0.06^*^	0.03	[0.00, 0.11]
	Grade				−0.04	0.04	[−0.11, 0.03]
	RD				0.13^***^	0.03	[0.07, 0.19]
	Self-esteem				−0.34^***^	0.04	[−0.42, −0.26]
**Effect**	**BJW values**	**B**		**Boot SE**	**Boot LLCI**		**Boot ULCI**
Indirect	*M*-SD (3.23)	0.04		0.02	0.01		0.07
	*M* (4.17)	0.07		0.01	0.04		0.09
	*M* + SD (5.11)	0.09		0.02	0.06		0.12

*N* = 1,261. RD, relative deprivation; BJW, Belief in a Just World. Bootstrap sample size = 5,000. CI, confidence interval; LL, low limit; UL, upper limit. ^*^p < 0.05, ^**^p < 0.01, ^***^p < 0.001.

**Figure 3:**
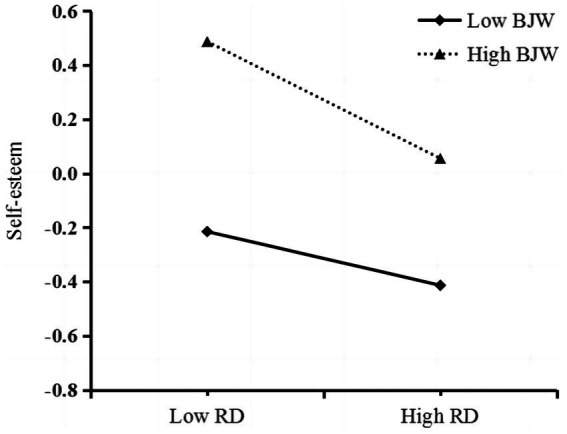
Moderating effect of relative deprivation on the prediction of self-esteem. RD, relative deprivation; BJW, belief in a just world.

In order to illustrate the moderating effect more clearly, we conducted a simple slope analysis separately for low (one SD below the mean) and high (one SD above the mean) levels of BJW (Aiken and West, 1991; see Figure 3). The diagram revealed that the effect of relative deprivation on self-esteem was more potent for migrant children with high levels of BJW (*simple slope* = −0.22, *t* = −5.75, *p* < 0.001). In contrast, the link between relative deprivation and self-esteem became weaker when migrant children had a lower BJW (*simple slope* = −0.10, *t* = −2.68, *p* < 0.01). In other words, the predictive effect of relative deprivation on self-esteem increased with an increase in BJW. The effect of relative deprivation on self-esteem was more powerful for migrant children with a higher BJW than for children with a lower BJW.

**Figure 4:**
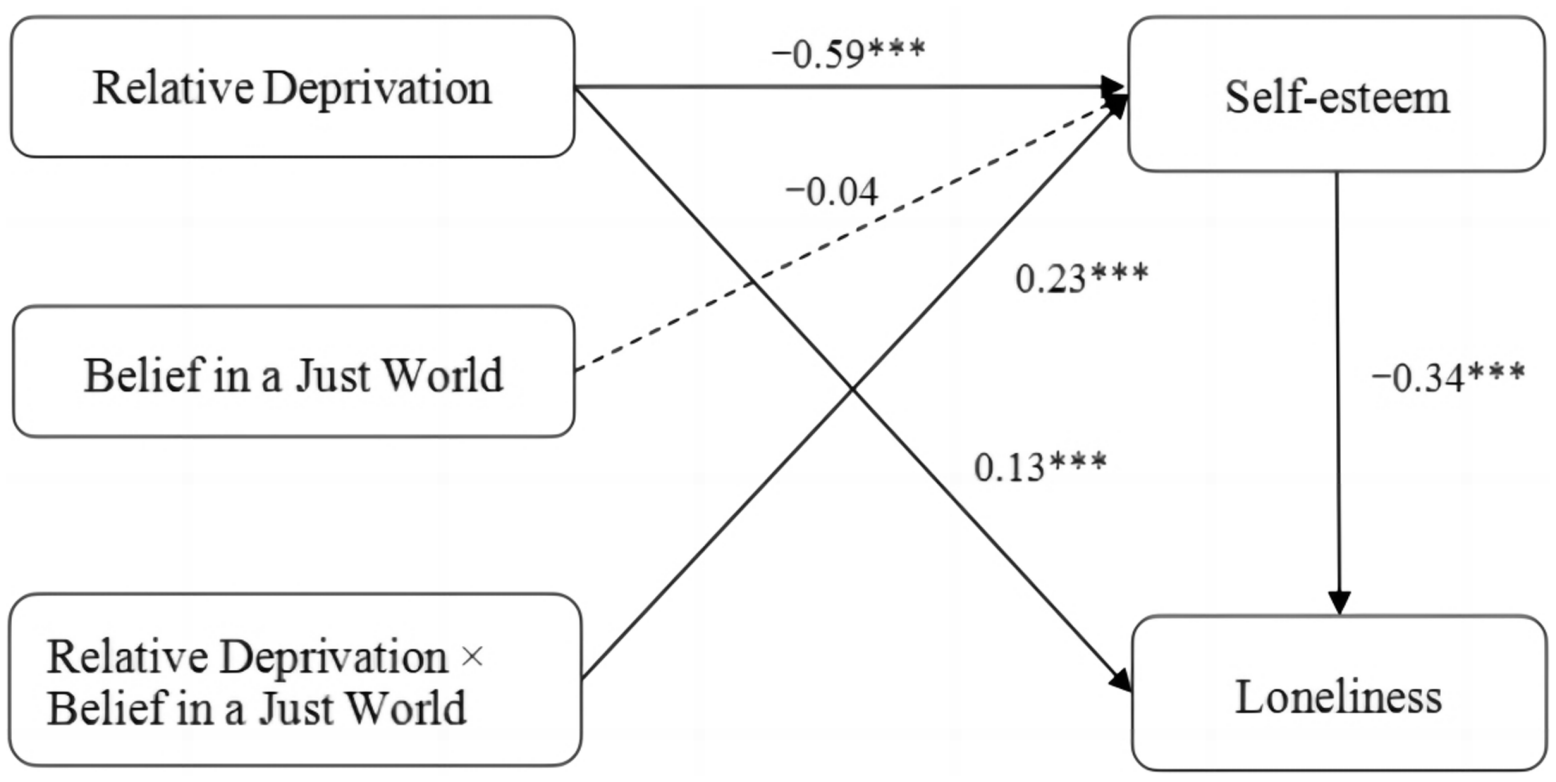
Moderated mediation model and key path coefficients for migrant children.

In conclusion, BJW intensified the effect of relative deprivation on loneliness, with the mediating effect of self-esteem being stronger for migrant children with high BJW. Thus, Hypothesis 3 was partially supported. Figure 4 depicts the moderated mediation model and key path coefficients for migrant children in detail.

### 4.4. Robustness test

To enhance the reliability and robustness of the empirical results and for the sample data to better explain the relationship between relative deprivation and loneliness, this study further tests the findings’ robustness by replacing the econometric model and reducing the sample size. (1) The substitution measurement model method (Tobit model) was adopted. Referring to Zhu’s (2020) research and other related studies, we used the Tobit model for regression estimation. As shown in Table 5, relative deprivation positively affects loneliness (LS) at the 1% level of significance, which is consistent with the original results and the main conclusion is still valid. (2) Next, we reduced the sample size (by 1%). By referring to Chen et al. (2022) research, we reduced the dependent variable (i.e., loneliness) by 1% at both ends and then re-estimated the benchmark model. After the sample size was changed *via* tail reduction, the test results remained consistent with the original results (see Table 6). This indicates that the results of the study are robust, that is, relative deprivation has a positive effect on the loneliness of migrant children.

**Table 5:** Robustness test: replacing the econometric model.

Variable	Loneliness
RD	0.209^***^
	(0.038)
Age	0.004
	(0.022)
Gender	−0.087
	(0.071)
Only-child status	0.029
	(0.088)
Grade	0.122^***^
	(0.022)
_cons	−0.411
	(0.296)
var(e. Loneliness)	1.235^***^
	(0.081)
*N*	1,261

RD, relative deprivation. Standard errors in parentheses. ^*^p < 0.05, ^**^p < 0.01, ^***^p < 0.001.

**Table 6:** Robustness test: narrowing the sample.

Variable	Loneliness
RD	0.195^***^
	(0.028)
Age	0.002
	(0.017)
Gender	−0.056
	(0.053)
Only-child status	0.024
	(0.066)
Grade	0.110^***^
	(0.017)
_cons	−0.293
	(0.223)
*N*	1261.000
*R* ^2^	0.092
*R*^2^_a	0.089

RD, relative deprivation. Standard errors in parentheses. ^*^p < 0.05, ^**^p < 0.01, ^***^p < 0.001.

**Figure 5:**
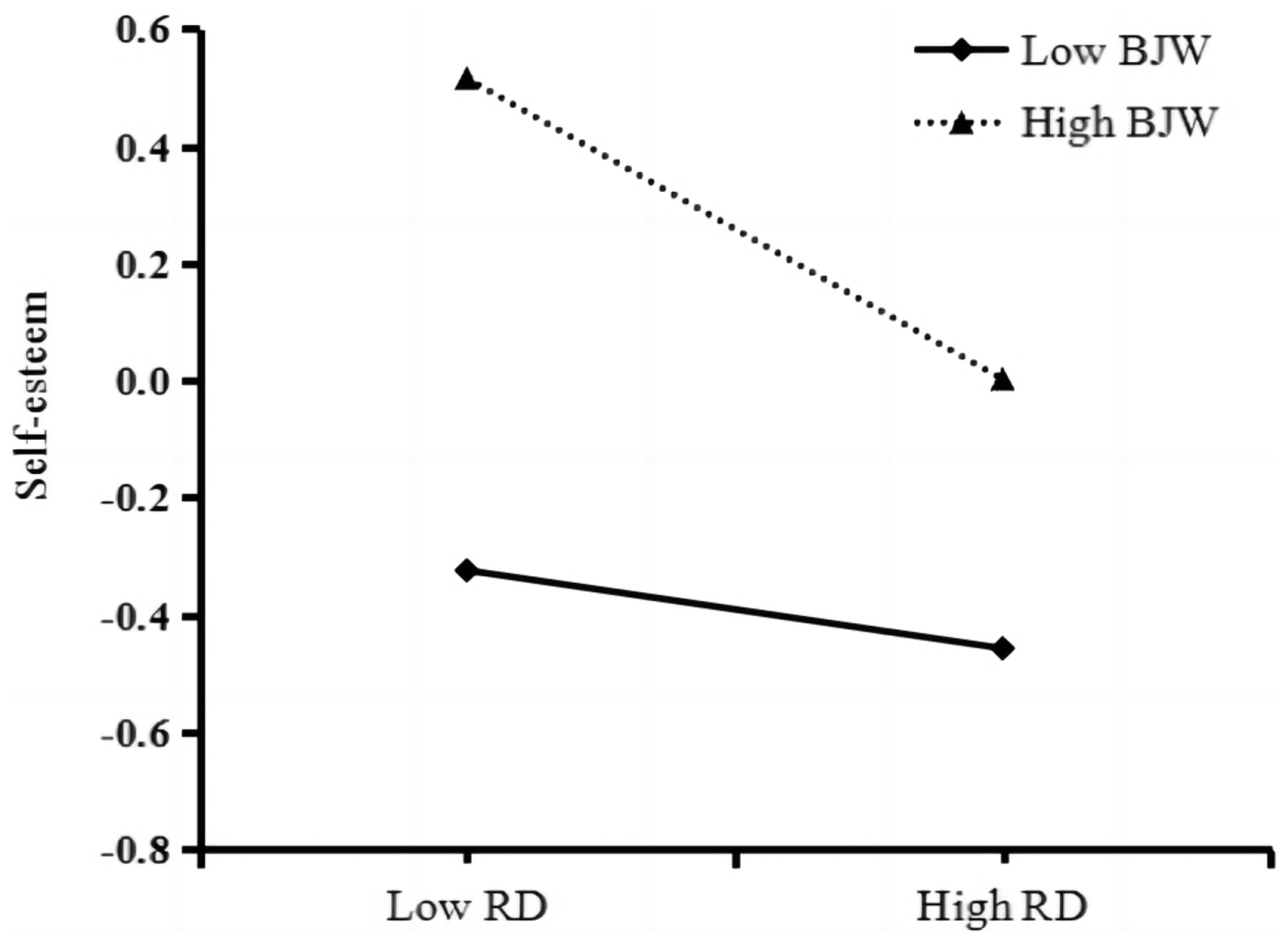
Robustness test: The moderating effect of relative deprivation on the prediction of self-esteem. RD, relative deprivation; BJW, belief in a just world.

Additionally, referring to Liao et al.’s (2022) research and other related studies, we conducted a robustness test on the unimputed sample (*N* = 773) using the PROCESS macro for SPSS software (Models 4 and 7; Hayes, 2013). The robustness test results reported in Tables 7, 8 support the previous results. As shown in Table 7, the direct relationship between relative deprivation and loneliness (*B* = 0.13, *t* = 3.69, *p* < 0.001) and the mediating effect of self-esteem (*B* = 0.09, *p* < 0.001, 95% CI: 0.06–0.12) remained significant. Therefore, the mediating role of self-esteem was confirmed. As shown in Table 8, the interaction between relative deprivation and BJW is significantly related to self-esteem (*B* = 0.22, *t* = 23.03, *p* < 0.001). In conclusion, BJW’s moderating effect on the relationship between relative deprivation and self-esteem is supported. The simple slope analysis in Figure 5 confirms that at a higher level of BJW, relative deprivation has a stronger predictive effect on self-esteem, compared with a lower BJW level. Therefore, the proposed mediation model was confirmed.

**Table 7:** Robustness test: testing the mediation effect of self-esteem.

Outcome (*Y*)	Predictors (*X*)	Model summary			*B*	SE	95% CI
		*R*	*R* ^2^	*F*			
Loneliness		0.32	0.10	16.93^***^			
	Age				0.02	0.02	[−0.02,0.07]
	Gender				0.03	0.07	[−0.11, 0.17]
	Only-child status				0.05	0.08	[−0.12, 0.22]
	Grade				0.10^***^	0.02	[0.06, 0.15]
	RD				0.21^***^	0.04	[0.14, 0.29]
Self-esteem		0.35	0.12	21.52^***^			
	Age				−0.01	0.02	[−0.05, 0.04]
	Gender				0.09	0.07	[−0.05, 0.23]
	Only-child status				0.06	0.08	[−0.10, 0.24]
	Grade				−0.14^***^	0.02	[−0.17, −0.09]
	RD				−0.23^***^	0.04	[−0.30, −0.16]
Loneliness		0.49	0.24	39.81^***^			
	Age				0.02	0.02	[−0.02, 0.06]
	Gender				0.07	0.06	[−0.06, 0.20]
	Only-child status				0.07	0.08	[−0.08, 0.23]
	Grade				0.05^*^	0.02	[0.01, 0.09]
	RD				0.13^***^	0.03	[0.06, 0.19]
	Self-esteem				−0.38^***^	0.03	[−0.45, −0.32]
**Effect**	***B***	**Boot SE**		**Boot LLCI**		**Boot ULCI**	
Direct	0.13	0.03		0.00		0.06	
Indirect	0.09	0.02		0.06		0.12	

*N* = 1,261. RD, relative deprivation. Bootstrap sample size = 5,000. LL, low limit; CI, confidence interval; UL, upper limit. ^*^p < 0.05, ^**^p < 0.01, ^***^p < 0.001.

**Table 8:** Robustness test: moderated mediation analysis results with BJW as a moderator.

Outcome (*Y*)	Predictors (*X*)	Model summary			*B*	SE	95% CI
		*R*	*R* ^2^	*F*			
Self-esteem		0.42	0.17	23.15^***^			
	Age				−0.01	0.02	[−0.05,0.04]
	Gender				0.09	0.07	[−0.04,0.23]
	Only-child status				0.02	0.08	[−0.14,0.19]
	Grade				−0.00	0.03	[−0.06,0.05]
	RD				−0.16^***^	0.04	[−0.24, −0.09]
	BJW				0.33^***^	0.05	[0.23,0.43]
	RD × BJW				0.22^***^	0.10	[0.21, 0.25]
Loneliness		0.49	0.24	39.81^***^			
	Age				0.02	0.02	[−0.02,0.06]
	Gender				0.07	0.06	[−0.06,0.19]
	Only-child status				0.07	0.08	[−0.08,0.23]
	Grade				0.05^*^	0.02	[0.01,0.09]
	RD				0.13^***^	0.03	[0.06, 0.19]
	Self-esteem				−0.39^***^	0.03	[−0.45, −0.32]
**Effect**	**BJW values**	**B**		**Boot SE**	**Boot LLCI**		**Boot ULCI**
Indirect	*M*-SD (3.23)	0.03		0.02	0.01		0.07
	*M* (4.17)	0.06		0.02	0.04		0.09
	*M* + SD (5.11)	0.10		0.02	0.06		0.12

*N* = 1,261. RD, relative deprivation; BJW, Belief in a Just World. Bootstrap sample size = 5,000. CI, confidence interval; LL, low limit; UL, upper limit. ^*^p < 0.05, ^**^p < 0.01, ^***^p < 0.001.

## 5. Discussion

The scale of China’s migrant population is expanding. Accordingly, the prevalence of mental health problems in this population should not be ignored, especially the occurrence of loneliness among migrant children. Migrant children are in a critical period of psychological development and require attention and protection (Chen et al., 2012; Pei and Liu, 2014). In the present study, we found that there were no differences in the loneliness of migrant children according to gender, only-child status, and grade; however, there was an overall rising trend of loneliness in tandem with age. The loneliness of migrant children aged 15 was significantly higher than that of other age groups. Having reached adolescence, 15-year-olds enter a peak period of loneliness. Studies have also shown that loneliness increases with age (Yang and Victor, 2011; Luhmann and Hawkley, 2016). Moderated mediation analysis showed that self-esteem partially mediated the association between relative deprivation and loneliness, and this effect was stronger for migrant children with a high BJW. These findings shed light on the potential mechanisms by which relative deprivation influences loneliness, while also providing insight into how to better help migrant children alleviate their loneliness and improve their mental health.

Consistent with previous studies, this study shows that relative deprivation has a positive predictive effect on loneliness. This finding supports social comparison theory. Migrant children perceive that they have fewer social support resources and social interaction resources through unreasonable social comparison with their peers, which induces loneliness and other negative emotional experiences (Fang et al., 2008). When migrant children follow their parents into urban life, their original social networks are broken, while new social networks take time to be set up in new cities and schools. This makes migrant children more concerned about their differences with their peers and more likely to develop a sense of relative deprivation (Xiong et al., 2021b). That is, they are more likely to perceive they are at a disadvantage compared to their peer (i.e., reference) group (Walker and Smith, 2002; Xiong and Ye, 2016). Loneliness is one of the main manifestations of this relative deprivation (Liesl and Eleonora, 2006; Cacioppo and Hawkley, 2009).

Relative deprivation not only positively predicted the loneliness of migrant children, but also influenced the loneliness of migrant children through the mediation of self-esteem. This finding is consistent with previous studies (e.g., Xiong, 2015) indicating that self-esteem is an important path for relative deprivation’s influence on the loneliness of migrant children. In this study, migrant children perceived themselves to be in a disadvantageous position, compared with their urban peers, and did not feel accepted by them. Such a perception leads to a lack of positive self-evaluation and low self-esteem (Fan et al., 2009; Xiong, 2015). Additionally, sociometer theory posits that migrant children are unable to receive positive comments to maintain and enhance their self-esteem under adverse social comparisons. Therefore, low self-esteem is not conducive for migrant children to maintain good social relations and develop a sense of group belonging. Thus, they feel a strong sense of loneliness (Leary, 2003; Leary, 2005; Zhang and Li, 2009). Self-esteem is an individual’s internal response to the sense of belonging to a group; thus, the lack of this sense of belonging enhances the individual’s sense of loneliness and affects their mental health (Leary et al., 1995b; Zhao et al., 2013; Waller, 2020). Overall, in the present study, self-esteem was identified as the underlying mechanism through which relative deprivation influenced migrant children’s loneliness.

Contrary to our hypothesis, BJW exacerbated the negative effects of relative deprivation on loneliness. Specifically, compared with migrant children with a low BJW, migrant children with a high BJW exhibited a stronger connection between relative deprivation and self-esteem. Contrary to our expectations, BJW did not alleviate the negative effect of relative deprivation on migrant children’s self-esteem. Instead, it intensified the negative impact of relative deprivation. Although studies have found that BJW may protect children from mental health problems (e.g., low self-esteem), especially relative deprivation (Otto et al., 2006; Alves and Correia, 2013; Tian, 2016), the results of our study and those of others (e.g., 김은하 et al., 2018; Kim and Park, 2018) do not support the protective effect of BJW. One possible explanation is that migrant children with high BJW may attribute negative situations (e.g., unfair treatment) to their abilities or internal weakness (Crocker and Major, 2003; Major et al., 2007), thus increasing the harmful effects of relative deprivation on self-esteem caused by negative social comparison.

These findings are also in line with the worldview verification model (Major et al., 2007; Townsend et al., 2010). Worldviews help human beings understand the social world’s basic demands and makes them feel valuable (Hogg, 2001; Fiske, 2004). Therefore, when the actual situation or their experience is consistent with the worldview (i.e., when the individual’s worldview is confirmed), the individual’s sense of certainty and security increases and positively impacts their development and growth. In contrast, when their worldview is threatened, the individual will experience pain, which is not conducive to individual growth (Greenberg et al., 1997; Kaiser et al., 2004; Lucas et al., 2016).

According to the worldview verification model, as migrant children with a low BJW believe that their world is unfair, their perception of relative deprivation confirms their worldview to a certain extent. On the contrary, relative deprivation threatens the worldview of migrant children with a high BJW. The power of a particular worldview determines the degree of negative effects experienced by the individual when such worldview is threatened. When the actual situation and experience are inconsistent with their worldview, individuals will experience a strong psychological threat. Therefore, in the case of relative deprivation, those with a high BJW will experience high self-esteem. In other words, migrant children who firmly believe that the world is fair and equitable are at a greater risk of being negatively affected by relative deprivation.

This study is helpful for people to better understand the relationship between relative deprivation and loneliness and its potential mechanisms. First, the results fit the sociometer theory and the worldview verification model. They also confirm the hypothesized moderating and mediating processes in the association between relative deprivation and loneliness (Major et al., 2007; Thomaes et al., 2010). On the one hand, it shows that self-esteem is a mediation mechanism between relative deprivation and loneliness among migrant children. On the other hand, BJW intensifies the negative effect of relative deprivation on self-esteem, while relative deprivation has a greater impact on the self-esteem of migrant children with high BJW. This confirms the different mechanisms of BJW in negative psychology, which aligns with the worldview verification model. Among them, it is noteworthy that BJW did not play a buffering role but aggravated the impact of relative deprivation on the loneliness of migrant children. These results may also be owed to culture. For instance, Chinese people generally believe that the world is just, orderly, and equal and that everyone gets what they deserve (Major et al., 2002; O’Brien and Major, 2005). However, in westernized capitalist countries, the dominant ideology is elitism. In other words, the differences in social status can be explained or proved according to the advantages and entitlement of individuals (e.g., *the American Dream*; Plaut et al., 2002; Major et al., 2007). When faced with a sense of relative deprivation, recognizing this elitism helps people rationalize unreasonable treatment and status differences, thereby helping maintain the stability of the status quo (Hafer and Olson, 1989; Major et al., 2007). In summary, this study provides a comprehensive understanding of how relative deprivation affects migrant children’s loneliness and the role of BJW in this relationship.

Additionally, some practical implications can be drawn from this study. First, we observed that relative deprivation positively affected migrant children’s loneliness and that this effect may increase with age. Therefore, schools, parents, and teachers should pay attention to the psychological adaptation and loneliness of migrant children as they enter high school and carry out educational interventions, such as thematic class meetings and weekly psychological notes (Hu, 2002). Second, self-esteem is an important mechanism in the relationship between relative deprivation and loneliness; thus migrant children themselves can enhance their self-esteem through self-referential tasks and psychological counseling and counseling services (Maricuoiu et al., 2019). Third, good social relationships help individuals feel recognized and accepted, as well as to form their own positive self-evaluations, thereby satisfying their need for self-esteem and reducing the experience of loneliness. Therefore, good parent–child relationships and harmonious teacher-student relationships facilitated by parents, teachers, and other elders serve as psychological support for migrant children. Conversely, integration groups for migrant children and non-migrant children can be established to encourage both sides understand and accept each other, as well as to help migrant children rebuild their social networks and enhance their sense of belonging (Zhang and Gai, 2012). Fourthly, we think it is necessary to carry out cognitive interventions on migrant children’s BJW. However, BJW is a double-edged sword. A very high level of BJW may affect migrant children’s understanding of the real world and lead to a strong sense of psychological threat. It may also make individuals more indifferent to their own predicaments and less likely to actively participate in changing them. On the contrary, a very low level of BJW is detrimental to an individual’s outlook and values. Therefore, it is necessary for schools, teachers, and parents to integrate dialectical thinking into the daily learning of migrant children, guide them in facing the real world, establish a correct concept of fairness, avoid treating justice and injustice from an isolated and one-sided perspective, and change their unfavorable situation by stimulating and mobilizing their subjective initiative. Additionally, a good social, school, and family environment can provide strong support for migrant children’s mental health. Therefore, the government and society should also pay attention to migrant children. Through policy guidance, life guidance, and grid management, it is possible to establish a good social atmosphere, improve care for the migrant population, and promote the formation of a positive and fair worldview among migrant children (Liao, 2018; Chen, 2019).

Despite the theoretical and practical implications discussed above, the current study had several possible limitations that warrant further exploration and research in the future. First, this research was conducted in a special group (i.e., migrant children), and there may be some applicability problems when extending it to other groups. We hope that different sample groups can be studied in future studies, given the different opinions on the moderating effect of BJW in other studies (Dalbert, 1999; Major et al., 2007; Sadiq and Bashir, 2015; Kim and Park, 2018), different ages and groups should be investigated. Second, given the contradictory results of different studies on BJW and its moderating role, future research can divide BJW into more detailed factors, such as personal belief in justice and social justice or individual and general BJW. Given the different dimensions of BJW and the various theoretical concepts involved, future research should explore the effect of different BJW dimensions on individual mental health and the moderating effect between relative deprivation, self-esteem, and mental health. Third, there may be other relevant variables that can affect the relationship between relative deprivation and loneliness among migrant children. Future studies could examine other related variables to explore whether BJW also has an impact on the relationship between relative deprivation and loneliness of migrant children, and whether there are other related variables that affect the mental health of migrant children. Fourth, as this was a cross-sectional study, it is difficult to draw inferences regarding causality. Future research in this direction should use longitudinal or experimental methods to clarify the relationship between relative deprivation and loneliness, especially the influence of self-esteem and BJW.

## 6. Conclusion

The current study expands the current understanding of the mechanisms underlying the effect of relative deprivation on migrant children’s loneliness by examining the mediating role of self-esteem and the moderating role of BJW. Specifically, the results show that after controlling for age, gender, only-child status, and grade, relative deprivation was significantly and positively correlated with migrant children’s loneliness, and this connection could be mediated by self-esteem. Moreover, the first part of the indirect effect of self-esteem on this link was moderated by BJW. These effects were stronger for migrant children with higher levels of BJW. This study reminds us that we should pay attention to the mental health problems of migrant children and implement corresponding intervention measures, particularly for those who experience loneliness.

## Data availability statement

The raw data supporting the conclusions of this article will be made available by the authors, without undue reservation.

## Ethics statement

The studies involving human participants were reviewed and approved by Ethics Committee of Academic Research at Yangtze University. Written informed consent to participate in this study was provided by the participants’ legal guardian/next of kin.

## Author contributions

MX conceived and designed the study, performed the survey, and authored and reviewed drafts of the manuscript. WX analyzed the data, prepared figures and tables, and wrote it into the article. All authors contributed to the article and approved the submitted version.

## Funding

This research was supported by a grant from the Key Project of Educational Science Planning of Hubei Province (no. 2022GA028) in China.

## Conflict of interest

The authors declare that the research was conducted in the absence of any commercial or financial relationships that could be construed as a potential conflict of interest.

## Publisher’s note

All claims expressed in this article are solely those of the authors and do not necessarily represent those of their affiliated organizations, or those of the publisher, the editors and the reviewers. Any product that may be evaluated in this article, or claim that may be made by its manufacturer, is not guaranteed or endorsed by the publisher.
